# Estrogen Regulates Estrogen Receptors and Antioxidant Gene Expression in Mouse Skeletal Muscle

**DOI:** 10.1371/journal.pone.0010164

**Published:** 2010-04-13

**Authors:** Kristen A. Baltgalvis, Sarah M. Greising, Gordon L. Warren, Dawn A. Lowe

**Affiliations:** 1 Department of Biochemistry, Molecular Biology, and Biophysics, Medical School, University of Minnesota, Minneapolis, Minnesota, United States of America; 2 Department of Physical Medicine and Rehabilitation, Medical School, University of Minnesota, Minneapolis, Minnesota, United States of America; 3 Division of Physical Therapy, Georgia State University, Atlanta, Georgia, United States of America; Istituto Dermopatico dell'Immacolata, Italy

## Abstract

**Background:**

Estrogens are associated with the loss of skeletal muscle strength in women with age. Ovarian hormone removal by ovariectomy in mice leads to a loss of muscle strength, which is reversed with 17β-estradiol replacement. Aging is also associated with an increase in antioxidant stress, and estrogens can improve antioxidant status via their interaction with estrogen receptors (ER) to regulate antioxidant gene expression. The purpose of this study was to determine if ER and antioxidant gene expression in skeletal muscle are responsive to changes in circulating estradiol, and if ERs regulate antioxidant gene expression in this tissue.

**Methodology/Principal Findings:**

Adult C57BL/6 mice underwent ovariectomies or sham surgeries to remove circulating estrogens. These mice were implanted with placebo or 17β-estradiol pellets acutely or chronically. A separate experiment examined mice that received weekly injections of Faslodex to chronically block ERs. Skeletal muscles were analyzed for expression of ER genes and proteins and antioxidant genes. *ERα* was the most abundant, followed by *Gper* and *ERβ* in both soleus and EDL muscles. The loss of estrogens through ovariectomy induced *ERα* gene and protein expression in the soleus, EDL, and TA muscles at both the acute and chronic time points. *Gpx3* mRNA was also induced both acutely and chronically in all 3 muscles in mice receiving 17β-estradiol. When ERs were blocked using Faslodex, *Gpx3* mRNA was downregulated in the soleus muscle, but not the EDL and TA muscles.

**Conclusions/Significance:**

These data suggest that *Gpx3* and *ERα* gene expression are sensitive to circulating estrogens in skeletal muscle. ERs may regulate *Gpx3* gene expression in the soleus muscle, but skeletal muscle regulation of *Gpx3* via ERs is dependent upon muscle type. Further work is needed to determine the indirect effects of estrogen and *ERα* on *Gpx3* expression in skeletal muscle, and their importance in the aging process.

## Introduction

There has been debate as to whether or not estrogen affects the force-generating capacity of skeletal muscle. A recent meta-analysis was conducted by our lab examining 23 studies in which postmenopausal women who were and were not taking estrogen hormone replacement were subjected to tests of muscle strength [1]. Our findings demonstrated a significant effect of approximately 5% greater strength in women who were taking estrogen replacement therapy. These findings are corroborated by a recent study that examined muscle strength in twins, in which one twin took estrogen replacement, and the other did not [2]. This paper showed that the hormone replacement therapy users walked at a maximal speed faster than non-users, and they had greater muscle power. We have extended the findings in humans using a mouse ovariectomy model. We showed that muscle and myosin functions were reduced ∼20% in ovariectomized mice [3], and that those losses in force generation at both the whole muscle and molecular levels were completely restored when mice were administered 17β-estradiol [4].

How estradiol confers its beneficial effects to skeletal muscle and contractile proteins is not known. Theoretically, it could happen by non-genomic or genomic mechanisms. The most well-described mechanism for estradiol action in reproductive tissue is its genomic effects that are mediated through estrogen receptors (ER). In skeletal muscle, two isoforms of ERs have been identified, estrogen receptor α (ERα or Esr1) and estrogen receptor β (ERβ or Esr2). These have been identified in multiple species including mice [5] and humans [6]–[9]. In human skeletal muscle, *ERα* mRNA levels are not different between males and females [6] and are expressed 180-fold greater than *ERβ* mRNA [7]. More recently, ERα and ERβ protein have been detected in human muscle, with about 2/3 of myonuclei staining positive for the receptors [7], [9]. Less work has been done in mouse skeletal muscle. *ERα* mRNA was detected in mouse skeletal muscle, but *ERβ* mRNA levels were undetectable [5]. In addition to ERα and ERβ, a third isoform of the estrogen receptor, G-protein coupled receptor 30 (Gper or GPR30), has been recognized in several tissues, but is minimally expressed in skeletal muscle [10], [11] and satellite cells [12].

Most of the work that has been done to elucidate a role for the different ERs in muscle has been conducted in cell culture. Both ERα and ERβ have been carefully characterized for their localization in C2C12 cells [13], [14]. Human skeletal muscle cells treated with estrogen increase steroid receptor coactivator (*SRC*) and decrease silencing mediator for retinoid and thyroid hormone receptors (*SMRT*) mRNAs, suggesting transcriptional activity of the ER in response to estradiol [15]. Many potential ER-dependent mechanisms in myoblasts have been investigated, including their role in stimulating the PI3K/Akt pathway [16], Glut-4 expression [15], muscle differentiation via upregulation of myogenin and myosin heavy chain [17], MyoD activity [18], and prevention of apoptosis [19], [20]. While studying the effects of estrogen and ERs in culture warrants merit and has yielded important information, intact skeletal muscle is composed of fused myotubes, is multi-nucleated, and innervated. A physiological consequence of estrogen deprivation in this complex environment is a reduction in the force-generating capacity of muscle in both women and rodents. Therefore, investigating the role of ER-dependent mechanisms *in vivo* is necessary to elucidate the mechanisms by which myosin is affected, and ultimately to understand the contractile dysfunction that occurs in estrogen-deficient, aged women.

A link between estradiol-induced changes in ER expression and muscle function is likely complicated. While the downstream targets of the ER are many, estrogen-responsive genes that regulate oxidative stress are interesting to consider. Ovariectomized mice have lower levels of many antioxidant enzymes in the heart, including glutathione peroxidase, catalase, and superoxide dismutase [21], [22]. Mitochondria from the liver and brain of ovariectomized mice also produce more H_2_O_2_ [23]. However, these decrements are ablated when estrogen is replaced. These studies strongly indicate that estradiol plays an important role in balancing oxidative stress in non-skeletal muscle tissues. In skeletal muscle, balancing oxidative stress is crucial for myosin function [24] and overall muscle contractility, particularly during aging [25]. Elucidating a role for ERs in skeletal muscle most likely involves linking the ER with both estrogenic and aging effects, making antioxidant pathways worth investigating.

In summary, the literature illustrates that ERs exist in skeletal muscle, but whether or not they display typical steroid receptor responsiveness to their ligand, i.e., estradiol, has not been studied in intact skeletal muscle. The primary purpose of this study was to test the hypothesis that *ERα*, *ERβ*, and *Gper* in skeletal muscle are responsive to changes in circulating estradiol. We also began work to try to understand how estrogen and/or ERs confer a benefit to skeletal muscle function. Thus, a secondary purpose of this study was to test the hypothesis that antioxidant gene expression in skeletal muscle is responsive to changes in circulating estradiol. In order to probe for a link between ERs and antioxidant function, we systemically blocked ERs using the ER antagonist, Faslodex. We hypothesized that blocking ERs would have a detrimental effect on antioxidant gene expression, providing a ER-dependent mechanism for estrogen function in skeletal muscle.

## Methods

### Mice and estrogen manipulations

Four-month-old female C57BL/6 mice were acquired from Jackson Laboratories (Bar Harbor, ME). Mice were group housed and had access to phytoestrogen-free food (Harlan-Teklad; #2019) and water ad libitum. The room was maintained on a 12∶12 light:dark cycle. The ovariectomy procedure [3] and 17β-estradiol pellet implantation procedure [4] were performed as previously described. Three experiments were performed: (1) ovariectomy and acute replacement of 17β-estradiol for 48 hours, (2) ovariectomy and chronic replacement of 17β-estradiol for 3 weeks, and (3) inhibition of ERs for 1 month with Faslodex.

In the acute study, after 7 days of estrogen withdrawal, mice received a placebo pellet (OVX + Placebo; *n* = 6) or a 17β-estradiol pellet (OVX + E_2_; *n* = 6) containing 0.18 mg of 17β-estradiol in a matrix that is designed to release the hormone (or placebo) over a 60-day period (Innovative Research of America, Sarasota, FL). Sham operations were performed (Sham; *n* = 4) on an additional group of mice. All mice were returned to individual cages following the subcutaneous pellet implantation and remained there for 48 hours. In the chronic study, ovariectomies were performed and mice were implanted with placebo or 17β-estradiol pellets (*n* = 6 per group) for a total duration of 3 weeks. This time point was chosen since we previously reported that skeletal muscle contractile dysfunction occurs between 3 and 4 weeks following ovariectomy [3], [4], [26].

In the third study, mice were injected weekly with the ER antagonist ICI 182,780 (Faslodex®, AstraZeneca) at a dose of 10 mg/kg body mass, or a similar volume of mineral oil (*n* = 5 per group) for a duration of 1 month. A daily dose of approximately 0.1 to 3 mg/kg BM has previously been shown to reduce uterine mass in mice and rats [27]–[29]. Mice were placed in voluntary activity wheels for 3 days before the start of injections, as well as 2 weeks following treatment, as an indicator of physical activity. The total amount of voluntary physical activity was averaged over the 3 days at each time point. Food intake and body mass was also monitored on a weekly basis.

At each study's end, mice were anesthetized with sodium pentobarbital (100 mg/kg body mass). Blood was collected by facial vein bleeds and plasma was stored at −80°C for the acute study. Uterine masses were recorded for the chronic study and the Faslodex study to ensure the validity of the ovariectomies and effective blocking of ERs. The soleus and extensor digitorum longus (EDL) muscles were dissected and immediately processed for RNA isolation. Tibialis anterior (TA) muscles were also dissected, snap frozen in liquid nitrogen, and stored at −80°C. All animal procedures were approved by the University of Minnesota's Institutional Animal Care and Use Committee.

### Circulating estradiol

An ELISA was used to measure 17β-estradiol in the plasma according to the manufacturers' specifications (KA0234, Abnova Corporation, Taiwan). The sensitivity of the assay was 10 pg/mL. All samples were run in duplicate. Standards were graphed and fit using a 4-parameter logistic curve fit. Plasma estradiol levels in sham-operated mice averaged 20±20 pg/ml. Circulating estradiol levels were below the level of detection (10 pg/ml) in all OVX + Placebo mice, while OVX + E_2_ mice had an average of 246±68 pg/mL.

### RNA isolation and DNase treatment

RNA was isolated using Tri Reagent® (Molecular Research Center, Inc., Cincinnati, OH) according to manufacturers' instructions. Briefly, muscles were dissected and immediately homogenized in 1 ml of Tri Reagent® on ice using a PowerGen homogenizer using 4×5 s pulses. Samples were incubated at room temperature for 5 minutes to allow the dissociation of RNA/protein complexes. Phase separation was achieved using 100 µL of bromochloropropane and vigorously shaking samples for 15 s. Samples were allowed to sit at room temperature for 15 minutes, followed by centrifugation at 12,000 g for 15 min at 4°C. The supernatant was transferred to a new tube, followed by precipitation with 500 µL of isopropanol. Samples were incubated for 10 min at room temperature and centrifuged at 12,000 g for 8 min at 4°C. The supernatant was discarded, the pellet was washed in 75% EtOH, and the tubes were centrifuged at 7,500 g for 5 min at 4°C. The pellet was briefly allowed to air dry and was resuspended in 100 µL of RNase-free H_2_O, and heated at 55°C for 10 minutes to help solubilize the RNA. RNA was purified and concentrated using the RNeasy® MinElute® Cleanup Kit (Qiagen, Valencia, CA) according to the manufacturers' instructions. The RNA quantity was determined by reading 1 µL of RNA using a NanoDrop ND-1000 spectrophotometer (Thermo Fisher Scientific, Wilmington, DE) at wavelengths 260 and 280. All 260/280 ratios were ≥2.0. One or 2.5 µg of RNA was diluted to a total volume of 8 µL and was treated with 1 µL of RQ1 RNase-free DNase (Promega, Madison, WI) and 1 µL of DNase buffer at 37°C in a thermal cycler (Techne, Model TC-312, UK) for 30 min. The reaction was terminated using 1 µL of stop solution and incubating the samples at 65°C for 15 minutes.

### cDNA synthesis and PCR Array

cDNA was made using a RT^2^ First Strand cDNA kit from SABiosciences (Frederick, MD). Briefly, 1 µg of DNase-treated RNA in a volume of 10 µL was added to 4 µL of RT buffer, 1 µL of Primer and External Control Mix, 2 µL of RT Enzyme Mix, and 3 µL of RNase-free H_2_0. This 20 µL cocktail was incubated for 15 minutes at 42°C in a thermal cycler. The reaction was terminated by incubating the samples at 95°C for 5 minutes. The tubes were then diluted with 91 µL of sterile ddH_2_O to have a final volume of 111 µL of diluted cDNA, and stored at −20°C until the PCR arrays were run.

Oxidative stress and antioxidant defense PCR arrays (PAMM-065) from SABiosciences were prepared for OVX + Placebo (n = 6) and OVX + E_2_ (n = 6) for both soleus and EDL muscles. One PCR array was used per sample. Each well on the plate was coated with primers specific for each gene. The PCR cocktail contained 1275 µL of 2x SA Biosciences RT^2^ master mix (containing SYBR® green dye), 102 µL of diluted cDNA, and 1173 sterile ddH_2_O for a total volume of 2550 µL; 25 µL of this cocktail was added to each well on plate. Plates were run on a Stratagene Mx3000P quantitative PCR system. The parameters on the machine included 1 cycle of 10 minutes at 95°C to activate the HotStart DNA polymerase. This was followed by a 2-step cycling program consisting of 40 cycles of a denaturing step of 15 sec at 95°C and an annealing step of 1 minute at 60°C. SYBR® green fluorescence was detected during the last 15 s of the annealing step. The cycle threshold (C_T_) was calculated automatically. The 2^∧^(-ΔΔC_T_) method was used for detecting changes in gene expression [30]. The ΔC_T_ was calculated by subtracting the average C_T_ for all 5 housekeeping genes (Gusb, Hprt1, Hsp90ab1, Gapdh, and Actb) from the gene of interest. Melting curves were run and no primer dimers were detected. Any wells having a C_T_ greater than 35 were considered undetectable. Control wells were also used for genomic DNA contamination, efficiency for reverse transcription, and a positive PCR control to ensure consistency between the individual plates. All of these values were within the range suggested by the manufacturer.

### cDNA synthesis and real-time PCR

One μg of DNase-treated RNA was used to synthesize cDNA using the High Capacity cDNA Reverse Transcription kit from Applied Biosystems (Foster City, CA). Briefly, 11 µL of DNase-treated RNA was added to a 9 µL cocktail containing 2 µL 10x RT buffer, 0.8 µL 25x DNTPs, 2 µL 10x random primers, 1 µL RT, 1 µL RNase-inhibitor, and 2.2 µL of RNase-free H_2_O. The mixture was incubated in a thermal cycler (Techne TC-312, UK) for 10 min at 25°C, 120 min at 37°C, 5 s at 85°C, and then held at 4°C overnight. Samples were diluted 1∶10 and 1∶100 in sterile ddH_2_O and stored at −20°C until real-time PCR analysis.

The Taqman® Gene Expression Assays for mouse-specific *ERα* (Mm00433149_m1), *ERβ* (Mm00599819_m1), *Gper* (Mm01194814_g1), *Gpx1* (Mm00656767_g1), *Gpx3* (Mm00492427_m1), *Nox4* (Mm00479246_m1), *Txnip* (Mm00452393_m1), *MyoD* (Mm01203489_g1), and *Glut-4* (Mm00436615_m1) were all purchased from Applied Biosystems (Foster City, CA). Eukaryotic 18S rRNA (4333760F) and β-actin (4352341E) were used as housekeeping genes. Each PCR reaction contained 9 µL of diluted cDNA,10 µL of 2x Taqman Master mix, 1 µL of 20x primer, for a total volume of 20 µL in each reaction. Samples were run in duplicate on ABI 7500 Fast Real-Time PCR System. The cycling conditions consisted of an initial step of 50°C for 2 minutes, followed by an initial denaturing step of 95°C for 10 minutes. The thermal cycler then followed a 2-step cycling program of denaturing at 95°C for 15 s and annealing at 60°C for 1 minute for a total of 40 cycles. FAM fluorescence was measured during last 15 s of the annealing step. The 2^∧^(-ΔΔC_T_) method was used for detecting changes in gene expression [30]. The ΔC_T_ was calculated by subtracting the average of the 18S rRNA and β-actin C_T_ values from the gene of interest. It should be noted that the housekeeping genes did not differ between treatment groups.

### Western blotting

Frozen TA muscle was homogenized in 1 ml of ice-cold RIPA buffer (50 mM Tris, pH 7.4, 150 mM NaCl, 1 mM EDTA, pH 8.0, 0.1% Triton-X, 0.1% SDS, 0.5% sodium deoxycholate, 5 µg/mL protease inhibitors (Sigma; P8340), and 10 µg/ml phosphatase inhibitors (Sigma; P5726). Homogenates were centrifuged at 10,000 g at 4°C for 10 min and the supernatants were stored at −80°C. ERα protein was measured by SDS-PAGE and Western blotting using an anti-mouse ERα (Clone MC-20, sc-542, Santa Cruz Biotechnology, Santa Cruz, CA) at a 1∶250 in Odyssey Blocking Buffer overnight at 4°C. Visualization of the antigen-antibody interactions were detected with the secondary antibody (Goat Anti-Rabbit IRdye® 680, LI-COR Biosciences, Lincoln, NE) at a dilution of 1∶500 in Odyssey Blocking Buffer. The membranes were then scanned and quantified using the Odyssey® Infrared Imaging System (LI-COR Biosciences).

### Data analysis

All gene and protein expression data were analyzed using student t-tests or one-way ANOVAs. Two-way ANOVAs were used to determine the effect of ER isoforms within muscle type, antioxidant gene expression within muscle type using the PCR array, and changes in body mass and wheel running activity over time. If a significant main effect or interaction existed, Tukey post-hoc tests were performed. Significance was set at P<0.05.

## Results

### *ERα*, *ERβ*, and *Gper* gene expression in skeletal muscle

**Figure 1:**
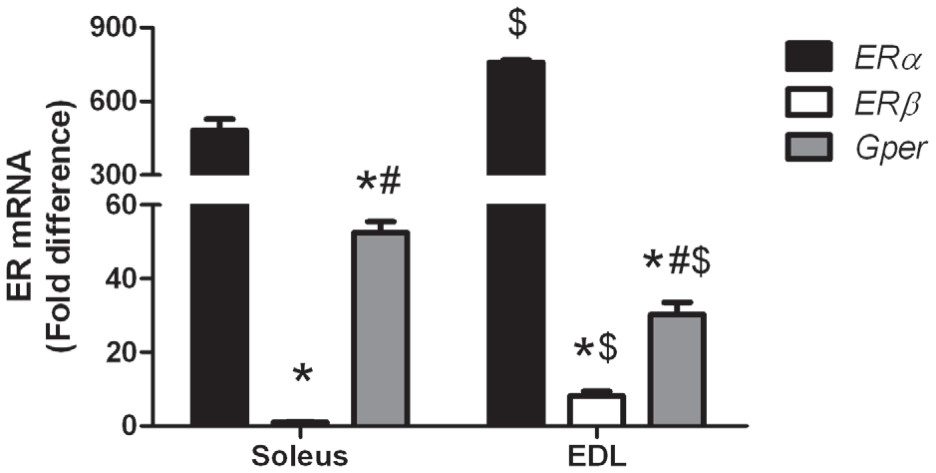
ER gene expression in skeletal muscles of 4-mo-old female wild-type mice. Data normalized to *ERβ* in the soleus. Values are means ± SEM. *Signifies different from *ERα* within a muscle type. ^#^Signifies different from *ERβ* within a muscle type. ^$^Signifies different from soleus muscle.

Real-time PCR was used to determine the relative abundance of *ERα*, *ERβ*, and *Gper* gene expression in female soleus and EDL muscles (Figure 1). In both muscles, *ERα* was the most abundant, followed by *Gper*, and *ERβ* was present in the least amount (P<0.001; Figure 1). We also directly compared ER gene expression between the slow-twitch soleus and fast-twitch EDL muscles (Figure 1). EDL muscles had 58% greater expression of *ERα* (P = 0.011) and 8-fold greater expression of *ERβ* (P<0.001) relative to soleus muscle. However, the soleus muscle had 74% greater expression of *Gper* (P = 0.003) compared to the EDL muscle.

### ER gene and protein expression are sensitive to ovariectomy and acute 17β-estradiol replacement

**Figure 2:**
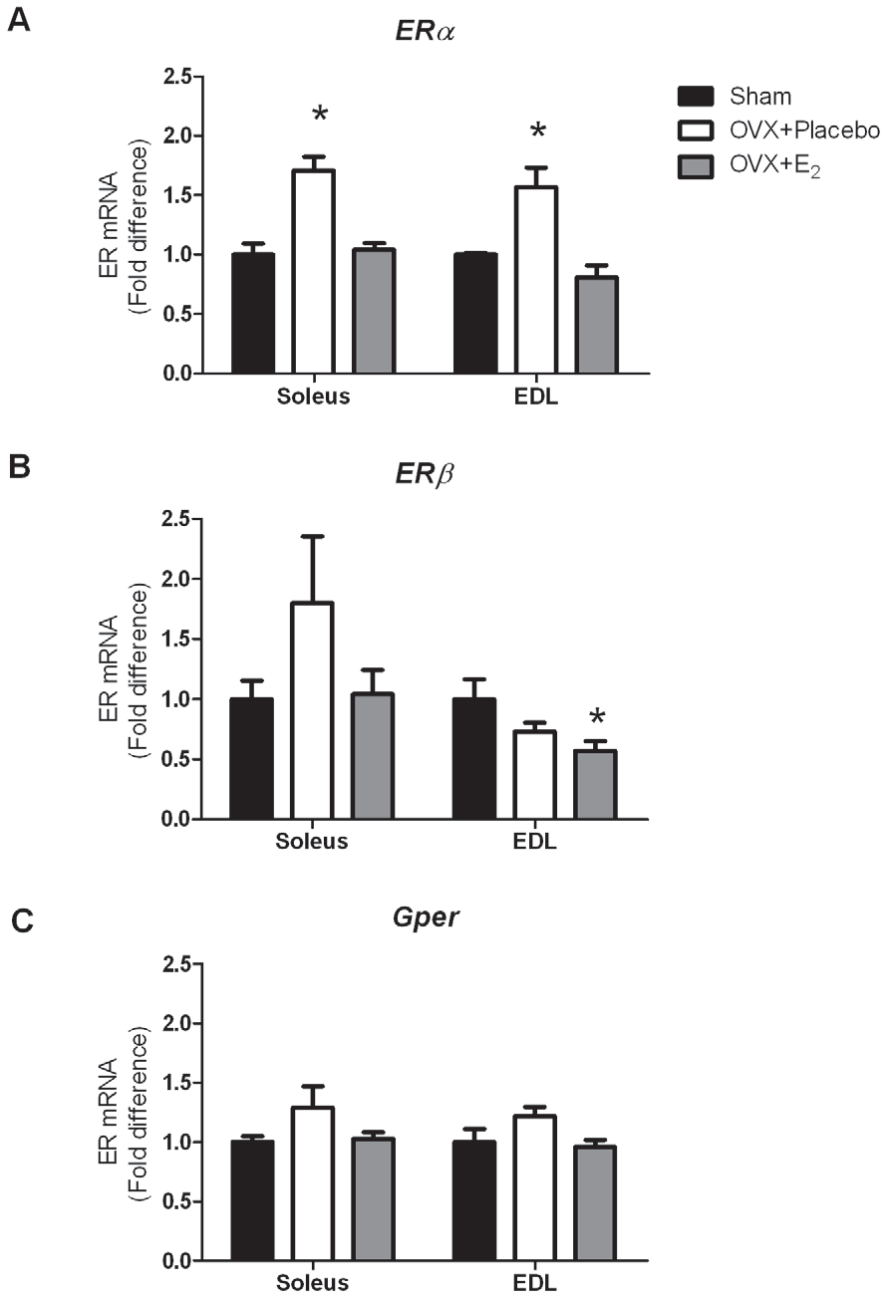
ER gene expression in skeletal muscle following ovariectomy and 48 hours of 17β-estradiol replacement. A. *ERα* gene expression. B. *ERβ* gene expression. C. *Gper* gene expression. Data are normalized to sham mice within each muscle. Values are means ± SEM. *Signifies different from sham. ^#^Signifies different from OVX + Placebo.

*ERα*, *ERβ*, and *Gper* mRNA levels were measured in the soleus and EDL muscles from sham-operated, OVX + Placebo, and OVX + E_2_ mice. There was a significant effect of estradiol status on *ERα* expression in both the soleus (P = 0.002) and EDL muscles (P<0.001). Ovariectomy resulted in muscle *ERα* mRNA being ∼70% greater compared to that in sham mice (Figure 2A). When 17β-estradiol was administered to ovariectomized mice, *ERα* mRNA levels came back down to sham levels in both muscles. *ERβ* mRNA levels were also altered in the EDL muscle (P = 0.038), with OVX + E_2_ mice having lower amounts of *ERβ* compared to sham mice in the EDL muscle. There were no effects on *Gper* expression in skeletal muscle in response to ovariectomy in either the soleus or EDL muscles (Figure 2C). Collectively, these data show that only *ERα* responds to acute changes in circulating 17β-estradiol in skeletal muscle.

**Figure 3:**
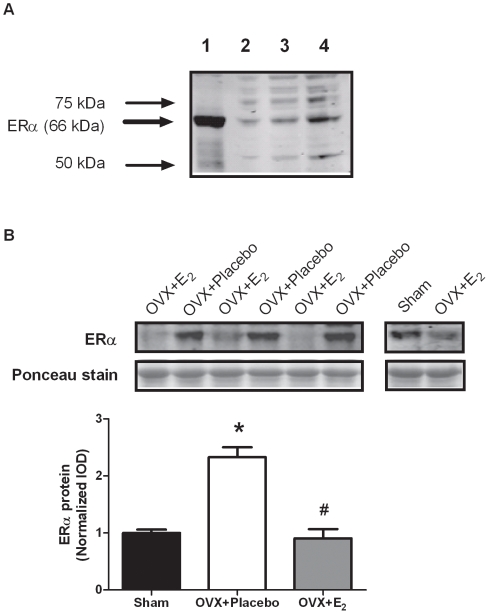
ERα protein expression in the TA muscle following ovariectomy and 48 hours of 17β-estradiol replacement. A. Preliminary work examining ERα expression in uterine tissue and skeletal muscle. Lane 1 = 10 µg of uterine homogenate. Lanes 2-4 = 10, 20, and 40 µg of skeletal muscle homogenate. B. ERα protein expression in muscle from sham, OVX + Placebo, and OVX + E_2_ mice. Data are normalized to sham mice. Values are means ± SEM. *Signifies different from sham. ^#^Signifies different from OVX + Placebo.

To determine if the changes in gene expression were representative of changes in protein expression, we examined ERα protein levels. To confirm that we were measuring ERα, we first qualitatively examined ERα protein levels in uterine tissue and skeletal muscle tissue, and also performed a dose-response curve in skeletal muscle. Figure 3A shows the relative large amount of ERα protein in uterine tissue compared to skeletal muscle, and also verifies that we are able to measure ERα protein in skeletal muscle. As shown in Figure 3B, ERα protein levels in the TA muscle responded in the same manner as did mRNA levels (P<0.001). Muscle from OVX + Placebo mice had about 2-fold more ERα protein than did those from OVX + E_2_ mice. Muscle from estradiol–replaced mice had similar ERα protein levels compared to those from sham mice.

### Acute 17β-estradiol replacement in ovariectomized mice induces antioxidant gene expression in skeletal muscle

One of the mechanisms by which estrogens may exert a beneficial effect on skeletal muscle is through its role as an antioxidant, or by activating ERs to regulate genes related to oxidative stress. We used PCR arrays to screen 84 genes related to the antioxidant defense system to determine if estradiol status was associated with regulation of these genes in skeletal muscle. We used a two-way ANOVA to compare the effect of treatment (placebo vs. 17β-estradiol) and muscle type (soleus vs. EDL) to determine global changes in skeletal muscle gene expression with estrogen. Our results show that 5 out of the 84 genes measured were greater in OVX + E_2_ mice compared to OVX + Placebo mice in both soleus and EDL muscles (Table 1; P≤0.027). These genes included *Gpx3*, *Gpx2*, *Nox4*, *Txnip*, and *Gpx1*. These data demonstrate that estrogen status can alter antioxidant gene expression in skeletal muscle.

**Table 1:** PCR array-determined antioxidant gene expression following replacement of 17β-estradiol in ovariectomized mice.

Gene	Muscle	Fold change	P-value
		(OVX+E_2_ vs. OVX+Placebo)	
*Gpx3*	soleus	3.85	<0.001
	EDL	2.19	
*Gpx2*	soleus	1.76	0.003
	EDL	1.31	
*Nox4*	soleus	1.71	<0.001
	EDL	1.46	
*Txnip*	soleus	1.49	<0.001
	EDL	1.18	
*Gpx1*	soleus	1.43	0.027
	EDL	1.46	

Values are means in fold difference from OVX + Placebo within each muscle type. The P-value represents the main effect of estradiol status.

### Chronic estradiol deprivation alters both ER expression and antioxidant gene expression

**Figure 4:**
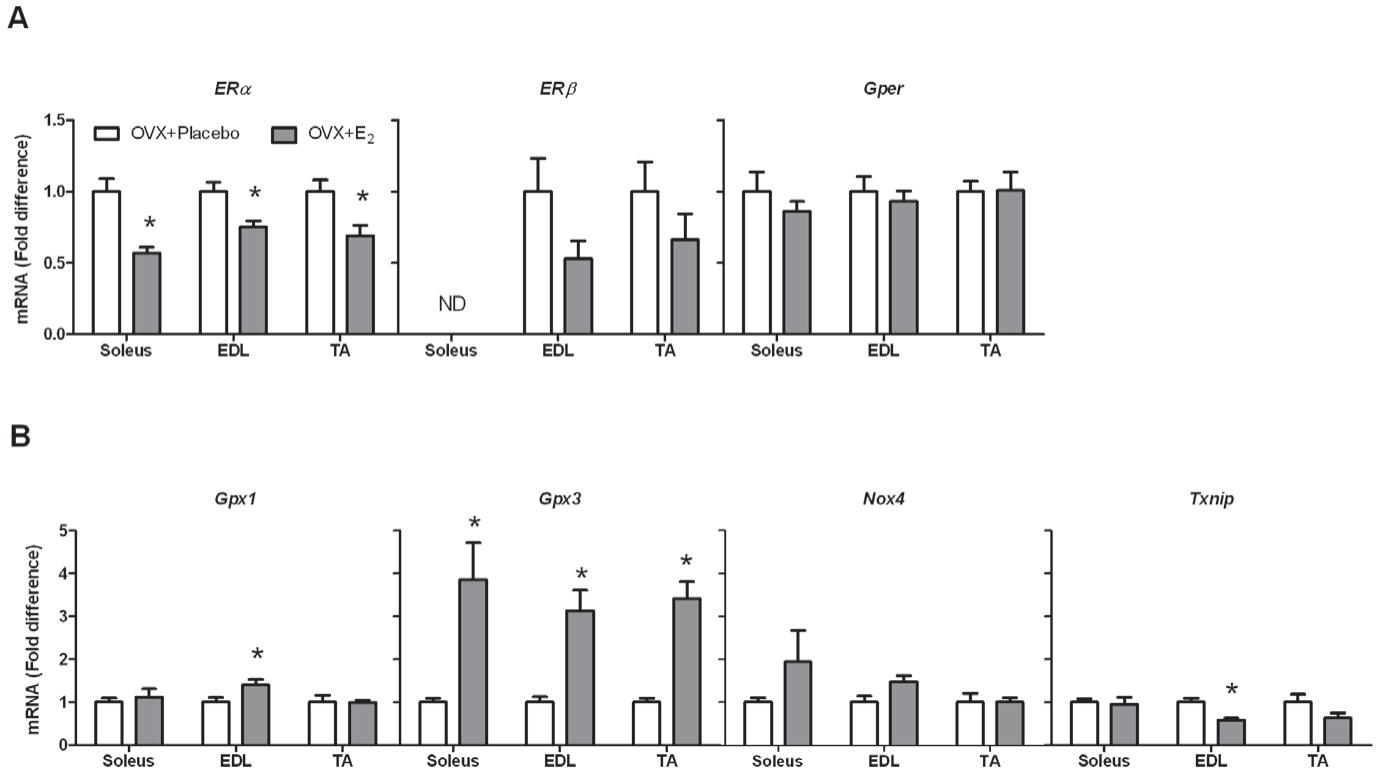
Chronic ovariectomy and 17β-estradiol replacement on ER and antioxidant gene expression in skeletal muscle. A. *ERα*, *ERβ*, and *Gper* gene expression. B. *Gpx1*, *Gpx3*, *Nox4*, and *Txnip* gene expression. ER and antioxidant gene expression were measured in the soleus, EDL, and TA muscles after 3 weeks of ovariectomy (OVX + Placebo) or in ovariectomized mice immediately replaced with 17β-estradiol (OVX + E_2_). Data are normalized to OVX + Placebo mice within each muscle. Values are means ± SEM. ND = not detected. *Signifies different from OVX + Placebo.

For ERs and antioxidant genes to have an impact on skeletal muscle function, these effects must be chronic in nature. Our next study was designed to examined ER and antioxidant gene expression in skeletal muscle following 3 weeks of ovariectomy with or without 17β-estradiol replacement. First, *ERα*, *ERβ*, and *Gper* gene expression were measured in the soleus, EDL, and TA muscles from estrogen-deficient and estrogen-replete mice. As shown in Figure 4A, *ERα* expression in OVX + E_2_ mice was 57%, 75%, and 69% of OVX + Placebo mice in the soleus, EDL, and TA muscles, respectively, similar to results from the previous acute study (P≤0.038). *ERβ* and *Gper* expression in skeletal muscle from this chronic study also mimicked the results from the acute study. Ovariectomized mice expressed similar amounts of ERβ and Gper in all muscles tested relative to those from mice replaced with 17β-estradiol. These data show that long-term deprivation of estradiol alters *ERα* gene expression in skeletal muscle.

Antioxidant gene expression was also quantified in these mice. Instead of screening 84 genes using the PCR arrays, individual PCR reactions were run for 4 out of the 5 genes that we found to be estradiol-responsive at the 48-hour time point: *Gpx3*, *Gpx1*, *Nox4*, and *Txnip*. *Gpx2* was not analyzed because it is classically defined as a gastrointestinal glutathione peroxidase [31]. Only *Gpx3* was responsive in all 3 muscle types following 3 weeks of ovariectomy and 17β-estradiol replacement (Figure 4B). *Gpx3* gene expression in 17β-estradiol-treated mice was 3.9-, 3.1-, and 3.4-fold greater, respectively, than that in ovariectomized mice (P≤0.001 for all muscles). There were also modest effects of *Gpx1* mRNA (P = 0.035) and *Txnip* mRNA (P = 0.003) in 17β-estradiol-treated mice, but this effect only occurred in the EDL muscle. These data suggest that *Gpx3* is positively regulated by 17β-estradiol in skeletal muscle.

### Inhibition of ER by Faslodex alters antioxidant gene expression

**Figure 5:**
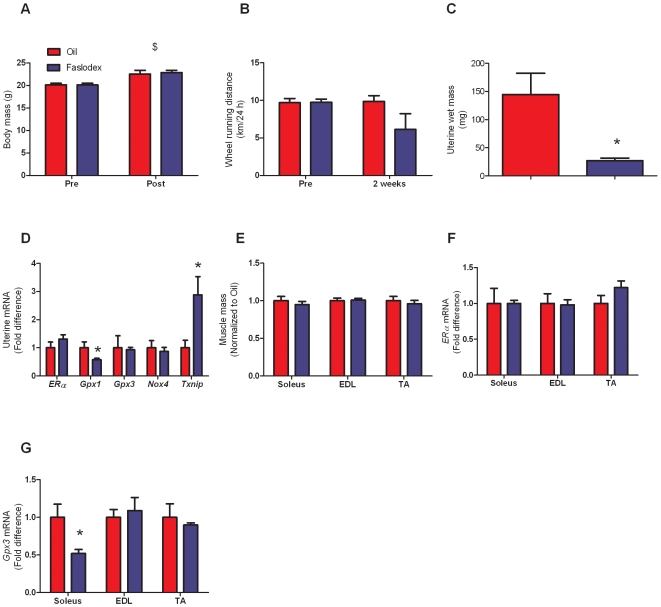
Effects of chronic ER inhibition on uterus and skeletal muscle. A. Body mass. B. Voluntary wheel running activity. C. Uterine wet mass. D. Uterine gene expression. E. Skeletal muscle mass. F. Skeletal muscle ER gene expression. G. Skeletal muscle *Gpx3* expression. ERs were blocked by administering Faslodex for 1 month to female mice. Data are normalized to oil-injected mice. Values are means ± SEM. *Signifies different from Oil. $Main effect of time.

Our next study was designed to determine if ERs regulate *Gpx3* expression in skeletal muscle. We treated mice for 1 month with Faslodex, an ER antagonist. There were no differences between oil and Faslodex-treated mice regarding body mass before or at the study's end, but both groups gained weight over the course of the study at the same rate (Figure 5A; P<0.001). Faslodex did not alter food intake by the mice (P≥0.651). There were no differences in acute wheel running activity between the 2 groups before the treatment, but there was a trend after treatment for Faslodex-treated mice to run 40% less than oil-injected mice (Figure 5B; P = 0.093). At the end of the study, uterine wet mass was 81% less in Faslodex-treated mice compared to mineral oil-injected control mice (Figure 5C; P = 0.015), validating that Faslodex did indeed block ERs. Although *ERα* gene expression in the uterus was unaltered with Faslodex, *Gpx1* was downregulated 58% (P = 0.046) and *Txnip* mRNA was upregulated 3-fold (Figure 5D; P = 0.016). Collectively these data validate that the Faslodex treatment was effective at blocking ERs to decrease uterine mass and alter antioxidant gene expression in that tissue.

Skeletal muscle mass, *ERα*, and *Gpx3* gene expression were measured in the soleus, EDL, and TA muscles after Faslodex treatment. Muscle mass was unaltered in all 3 muscles (P≥0.208; Figure 5E) as was ERα gene expression (P≥0.176; Figure 5F). There was a 50% decrease in *Gpx3* gene expression in the soleus muscle in response to Faslodex treatment (P = 0.019), but *Gpx3* levels remained the same in the EDL and TA muscles (Figure 5G).

### Estrogens minimally alter *MyoD* and *Glut-4* mRNA *in vivo*

**Figure 6:**
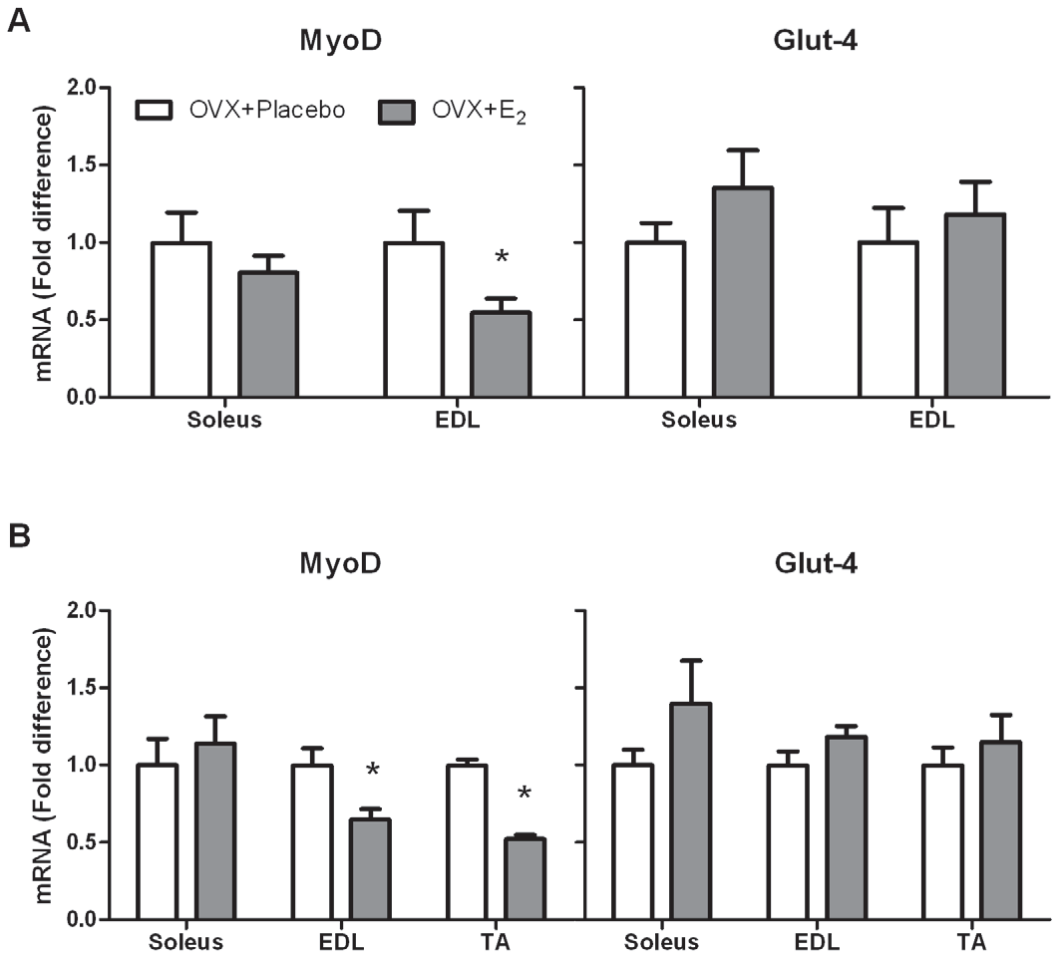
*MyoD* and *Glut-4* gene expression in ovariectomized mice with and without 17β-estradiol supplementation. A. *MyoD* and *Glut-4* after 48 hours of estrogen replacement. B. *MyoD* and *Glut-4* mRNA expression after 3 weeks of estrogen replacement. Values are means ± SEM. *Signifies different from OVX + Placebo.

Markers of muscle differentiation and hypertrophy, as well as glucose metabolism were also measured, since estrogen has robust effects on these markers in cell culture. We found that *MyoD* mRNA levels were reduced 45% after 48 hours (P = 0.046) in the EDL muscle with estrogen, but were unchanged in the soleus muscle (Figure 6A) compared to ovariectomized mice. Similar results were found after 3 weeks of replacement where estrogen-treated mice had *MyoD* mRNA levels that were ∼40% less in the EDL and TA muscles (P≤0.019), but *MyoD* was not affected in the soleus (Figure 6B). *Glut-4* mRNA levels were not altered in the soleus, TA, or EDL muscles by ovariectomy at any time point measured (Figures 6A & 6B).

## Discussion

The main findings from this study were that *ERα*, *Gper*, and *ERβ* are all expressed in skeletal muscle, but that only *ERα* is responsive to both acute and chronic changes in circulating estradiol. Acute and chronic changes in circulating estradiol also caused changes in *Gpx3* gene expression. *Gpx3* expression appeared to be regulated by ERs, but this was a muscle-specific response. These results are vital first steps in discovering estrogen-mediated mechanisms that influence skeletal muscle contractility. On the whole, this is an important topic because the decline in circulating estrogens with age has been associated with muscle weakness in women. These findings have been replicated in mice who have undergone ovariectomies to mimic the low circulating estrogen state and have been further extended to show that an underlying molecular explanation for the reduction in force generation involves myosin. However, it is unknown whether the effects of estrogen on muscle and myosin functions are via an ER-mediated mechanism. The current study begins to elucidate this mechanism by examining skeletal muscle ERs in estrogen deficient and -replete states.

While it is known that ERα and ERβ are present in skeletal muscle, much less is known about Gper. Our first experiment aimed to determine the relative mRNA abundance of all 3 ERs in skeletal muscle. Our findings are unique in that this is the first study to compare all three ER isoforms and their relative abundance in skeletal muscle. Previous work showed greater levels of *ERα* compared to *ERβ* mRNA [5], [7], and separate studies have reported the presence of *Gper* mRNA in skeletal muscle [32]. Our results complement these previous studies by reinforcing that *ERα* mRNA is the most abundant, and we also have added that *Gper* is expressed in a moderate amount, in between *ERα* and *ERβ*.

Our experimental design also allowed us to directly compare the relative mRNA abundance of these 3 ERs amongst slow-twitch (soleus) and fast-twitch (EDL) muscles. We found that *ERα* and *ERβ* were expressed in higher amounts in the EDL than soleus, but *Gper* was found in a greater amount in the soleus compared to the EDL. These data are somewhat contradictory to previously published findings. Lemione and coworkers reported that *ERα* mRNA was greater in the slow-twitch soleus muscle compared to the primarily fast-twitch gastrocnemius and EDL muscles in female rats [33]. ERα protein levels were also reported to be greater in the soleus compared to the gastrocnemius muscle in rabbits [34], [35]. The discrepancies for these findings could be based on many variables, including the species studied, age of the animals, and the methods of detection (e.g., only in our study was real-time PCR used). In addition, while the soleus is considered a slow-twitch muscle in the mouse, the percentage of fibers exhibiting Type I myosin heavy chain is only about 50%, compared to approximately 90% of the fibers in soleus muscles of the rat and rabbit [36]. Nevertheless, our data show that all three ERs are expressed at the gene level in skeletal muscle, with *ERα* being the most abundant, and having more expression in fast-twitch than slow-twitch muscle in female mice.

Our main objective was to determine how ERs in skeletal muscle respond to changes in circulating estrogen levels both acutely and chronically. This is the first report of changes in ER gene expression resulting from ovariectomy and 17β-estradiol replacement in skeletal muscle. Our data show that only *ERα* is responsive to 17β-estradiol status at both a 48-hour and 3-week time point. To corroborate our gene expression findings, we also examined ERα protein expression in the TA muscle, and protein expression mimicked gene expression. These data are also novel in that it is the first time ERα protein levels have been shown to be sensitive to acute changes in circulating estradiol in skeletal muscle. This places skeletal muscle in the category of an estrogen-sensitive tissue along with uterus, kidney, and cerebral cortex [37]. This work is also in agreement with others who have found that ovariectomy induces *ERα*, but not *ERβ* in white adipose tissue [38]. It is possible that we did not detect changes in *ERβ* with ovariectomy because the gene is expressed at such a low abundance in skeletal muscle. Failing to see a change in *Gper* gene expression does not rule it out as a possible important player in the maintenance of skeletal muscle function. *Gper* works in other cell types via signaling through MAPK and PI3K to induce gene transcription [39]. Therefore, estradiol-induced activation of these downstream signaling proteins via *Gper* could play a role in skeletal muscle function, and more investigation regarding the effects of *Gper* on skeletal muscle function are needed.

While it is novel to show changes in ER expression at both the gene and protein level with circulating estrogens *in vivo*, our next step was to begin to elucidate a role for ERs in skeletal muscle function. Since aging and the loss of estrogens both cause decrements in skeletal muscle function, and aging and estrogen deficiency are related to problems with antioxidant capacity, our next step was to see if antioxidant gene expression is altered in skeletal muscle with ovariectomy. We screened 84 antioxidant genes in skeletal muscles from ovariectomized mice 48 hours after treatment with 17β-estradiol or placebo. *Gpx3*, *Gpx2*, *Gpx1*, *Nox4*, and *Txnip* were increased in both the soleus and EDL muscles in ovariectomized mice replaced with 17β-estradiol. However, after 3 weeks of ovariectomy with or without estradiol treatment, only *Gpx3* gene expression was upregulated in response to the hormone. This held true for all 3 muscles examined, suggesting that it was a global skeletal muscle response. *Gpx3* is classically considered a plasma glutathione peroxidase, but also has high expression in the kidney, lung, brown adipose tissue, and white adipose tissue [40]. Females have a higher concentration of Gpx3 in the serum than males [41]. *Gpx3* can also be regulated by estrogen in white adipose tissue, with *Gpx3* mRNA being nearly 3-fold greater in ovariectomized mice treated with 17β-estradiol compared to vehicle-treated ovariectomized mice [38]. This effect is seen as early as 2 hours after treatment, and as long as 3 weeks in response to 17β-estradiol. Our data are in agreement with Lundholm *et al*. in that *Gpx3* also appears to be very sensitive to 17β-estradiol in skeletal muscle.

We further investigated whether 17β-estradiol's effect on *Gpx3* expression was regulated by ERs by blocking ER action with Faslodex. The treatment was successful since uterine masses were significantly lower in Faslodex-treated mice compared to oil-injected controls. The thioredoxin antioxidant system has previously been shown to be highly regulated by estrogen and Faslodex in the uterus, with 17β-estradiol decreasing and Faslodex increasing *Txnip*, a negative regulator of the thioredoxin antioxidant pathway [42]. In the current study, Faslodex increased *Txnip* expression in the uterus, complimenting previously published work. However, *Gpx3* gene expression was downregulated with Faslodex only in the soleus muscle. This data is in agreement with others who have published that *Gpx3* is responsive to estradiol in white adipose tissue, and this is mediated via ERα [38]. Our data suggests that ERs can regulate *Gpx3* gene expression, but this effect is dependent upon skeletal muscle type, since Faslodex did not alter *Gpx3* expression in the TA or EDL muscle. This result was somewhat surprising since 17β-estradiol had such a robust effect on *Gpx3* gene expression in all 3 muscles, and *ERα* gene expression also responded in a similar fashion in all 3 muscles. One explanation for the muscle difference could be the oxidative capacity of the muscle types. The soleus is a highly oxidative muscle, containing many mitochondria. In cell culture studies of muscle cells, ERα has been found primarily localized to the mitochondria [13]. This is in contrast to the TA and EDL which are relatively more glycolytic, and may have fewer mitochondria.

Since estradiol induced *Gpx3* gene expression in the TA and EDL, but this effect was not blocked by Faslodex, estrogen may also be working indirectly to induce *Gpx3* gene expression in these fast-type muscles. At this point, we can only speculate what this indirect effect may be. One of the consequences of ovariectomy is a reduction in physical activity. We and others have shown that ovariectomized mice that have access to voluntary activity wheels run approximately 90% less than ovary-intact mice, but running activity returns to normal with 17β-estradiol replacement [43]. ERα KO mice also demonstrate lower levels of physical activity [44]. In the current study, there was a non-significant 40% reduction in voluntary wheel running activity in mice treated with Faslodex. We should note that voluntary wheel running behavior was only monitored acutely so exercise would not be a confounding variable. Physical inactivity is associated with an increase in oxidative stress [45]. One might speculate that the potential small decrease in physical activity induced by Faslodex may not detrimentally affect *Gpx3* expression in the TA and EDL. In contrast, since ovariectomy causes a drastic reduction in physical activity, this stimulus may be enough to impact *Gpx3* expression in the TA and EDL. Only one study has examined the response of Gpx3 in the serum following an acute bout of exercise, and it did not change immediately after exercise [41]. More work is needed to determine the specific response of *Gpx3* to acute bouts and exercise training in skeletal muscle.

An alternative hypothesis regarding the role of estrogen and the estrogen receptor in skeletal muscle is the ability of estrogen to affect muscle differentiation. For example, *MyoD* and *Glut-4* have been previously shown to be highly sensitive to estrogen in muscle cells in culture, as both of these markers increase with estrogen at the mRNA and protein levels [15], [17]. Furthermore, following downhill running, satellite cell proliferation is activated to a greater extent in ovariectomized rats that receive estrogen, as measured by MyoD, Pax7, and BrdU-labeled nuclei [46], [47]. These papers suggest that estrogen and ERs are important in promoting satellite cell differentiation, which might also lead to the growth of muscle, and theoretically the subsequent strength gains seen in women and mice supplemented with estrogen. However, our data would suggest that MyoD and Glut-4 are not involved in estrogen's positive effect on skeletal muscle contractility. We did not detect changes in *Glut-4* mRNA levels with estrogen in any muscle or time point tested. In fact, *MyoD* levels actually decreased ∼50% with estrogen in the TA and EDL muscles at both time points. Our data are in line with the work of others who have examined glucose regulation [16], [48] and myogenic gene expression [49] in ovariectomized rats. Intact soleus muscles incubated with estrogen did not have enhanced glucose uptake, despite increased phosphorylation in upstream signaling proteins, such as Akt and AMPK [16]. Furthermore, basal levels of Glut-4 protein and glucose uptake are not affected by ovariectomy in rats [48]. Unlike the work done in cell culture, *MyoD* gene expression increased nearly 2-fold in the quadriceps muscle of estrogen-deficient mice compared to controls [49], nearly mimicking the results of our study. Since estrogen can regulate myogenic gene expression and promote differentiation *in vitro* [15], [17] and activate satellite cells after injurious exercise [46], [47], we speculate that the beneficial effects of estrogen on MyoD and Glut-4 expression may involve inducing satellite cells to undergo differentiation. This would be important during a period of muscle injury and recovery, but not necessarily contribute to the mechanism that underlies muscle weakness that occurs with aging and menopause. Our speculation is further substantiated by the fact that muscle fiber cross-sectional area, total protein content, and contractile protein content does not change with ovariectomy [3], [4], [50], [51] or in ERα^−/−^ and ERβ^−/−^ mice [52].

In summary, this study has many findings that have not been previously reported. First, we showed the relative abundance of the three ERs in skeletal muscle are, in decreasing order, *ERα*, *Gper*, and *ERβ*. Second, we demonstrated that only *ERα* is responsive to circulating estradiol levels at both acute and chronic time points. Third, *Gpx3* gene expression is highly sensitive to 17β-estradiol in skeletal muscle. Finally, the regulation of *Gpx3* by 17β-estradiol is possibly mediated via ERα in the soleus muscle, but also indirectly as consequence of ovariectomy, such as physical activity. Important future work is needed to determine the importance of *Gpx3* in skeletal muscle, and ultimately how this affects skeletal muscle function during aging.
